# Treadmill Exercise Training Ameliorates Apoptotic Cells and DNA Oxidation in the Cerebral Cortex of Rats Exposed to Chronic Ketamine Abuse

**DOI:** 10.1111/adb.70025

**Published:** 2025-03-10

**Authors:** Salar Sabziparvar, Kazem Khodaei, Javad Tolouei Azar

**Affiliations:** ^1^ Sport Physiology and Corrective Exercises Department, Sport Sciences Faculty Urmia University Urmia Iran

**Keywords:** addiction, apoptosis, drug abuse, exercise, ketamine, oxidative stress

## Abstract

**Background:**

Ketamine abuse damages brain function and structure, increasing reactive oxygen species and apoptosis in the cerebral cortex, but moderate‐intensity continuous training (MICT) can enhance antioxidant defences and reduce apoptosis. Therefore, we aimed to answer whether MICT can reduce the side effects of chronic ketamine abuse.

**Method:**

24 Wistar rats were split into control (CON), ketamine abuse (KET), exercise after ketamine withdrawal (KET + EX), and non‐intervention ketamine withdrawal (KET + WD) groups. Ketamine intervention groups received 50 mg/kg/day ketamine for 8 weeks; KET + EX underwent 5 MICT sessions/week at 60–75% VO2max for 8 weeks post‐withdrawal. Post‐sampling of cerebral cortex, we evaluated histological changes, apoptotic cell numbers, Bax, Bcl‐2, Caspase‐3 mRNA/protein, 8‐oxo‐2′‐deoxyguanosine (OXO) expression, glutathione peroxidase (GPX) and glutathione reductase (GR) mRNA and other oxidative stress and antioxidant markers levels. Effect sizes (ES) were used to assess group differences.

**Results:**

MICT significantly reduced apoptotic cells (ES = 14.24, *p* < 0.0001), decreased Bax and caspase‐3 protein expression, and increased Bcl‐2 compared to the KET group (Bax: ES = 2.77, *p* = 0.005; caspase‐3: ES = 7.73, *p* < 0.0001; Bcl‐2: ES = 12.11, *p* < 0.001). It also lowered Bax and caspase‐3 mRNA (Bax: ES = 4, *p* = 0.014; caspase‐3: ES = 2.29, *p* = 0.024). MICT reduced OXO and increased GR and GPX mRNA and nitric oxide (NO) level (GR: ES = 2.02, *p* = 0.016; GPX: ES = 1.98, *p* = 0.035; OXO: ES = 11.39, *p* < 0.0001; NO: ES = 3.52, *p* = 0.003). Levels of malondialdehyde, myeloperoxidase, glutathione, superoxide dismutase, and catalase remained unchanged between groups.

**Conclusion:**

MICT seems effective in reducing apoptosis and oxidative damage in the cerebral cortex of rats with long‐term ketamine abuse.

## Graphical abstract

Moderate‐intensity aerobic training (MICT) for 8 weeks resulted in a reduction in apoptosis indices such as TUNEL‐positive cells, Bax, Caspase‐3, increased Bcl‐2, and limited damage to cortical neuron. MICT has the ability to reduce apoptosis through BDNF/NO pathways. Apoptosis and DNA oxidation may be triggered by Ketamine abuse through glutamate/calcium influx, but ROS levels were not elevated. MICT decreased DNA oxidation and increased GR and GPX expression, while SOD, CAT, MPO, and MDA remained unchanged. MICT is capable of preventing brain damage caused by ketamine abuse, but further research is needed.

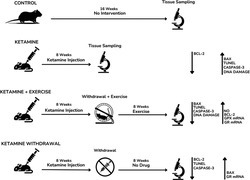

## 1 Introduction

Drug abuse is a global health problem which contributes to increased mortality rates [1]. Conversely, drug withdrawal may hinder return to normalcy due to CNS pathology [2]. In recent years, the abuse of ketamine has been on the rise worldwide, leading to the occurrence of neurotoxicity in individuals [1, 3, 4]. Ketamine, developed in 1962 as an anaesthetic, has gained recreational popularity in nightclubs due to its potential to cause intense psychosis at high doses [3, 5, 6].

Ketamine, is an N‐methyl‐D‐aspartate (NMDA) receptor antagonist [7], can elevate reactive oxygen species (ROS) by promoting intracellular calcium influx [8]. This increase in ROS can impair mitochondrial function, potentially triggering apoptosis [9]. Chronic ketamine administration has also been shown to increase ROS levels and apoptotic markers in rat brain tissue [10]. Two pathways, intrinsic and extrinsic, mediate cell apoptosis; both converge in the execution phase where caspase‐3 plays a key role. In the intrinsic pathway, the Bcl‐2/Bax ratio, involving anti‐apoptotic Bcl‐2 and pro‐apoptotic Bax, serves as an effective indicator of apoptosis [11]. Apoptosis can cause cell shrinkage and atrophy in brain regions [12]. The cerebral cortex is vital for addiction's onset and recovery, handling decision‐making, voluntary movements, and impulse control [13, 14]. Cerebral cortex injury leads to cognitive problems, including difficulty paying attention, reasoning, and decision‐making [13]. Damage to the prefrontal cortex has also been shown to increase drug craving and relapses [15].

Research indicates cerebral cortex atrophy in ketamine abusers [16, 17], yet no cure exists for ketamine‐induced neurotoxicity. However, non‐steroidal anti‐inflammatory drugs, anticholinergics, and antibiotics show beneficial effects [18]. Ketamine abstinence combined with environmental enrichment, notably exercise, can reduce cardiac and renal toxicity in rodents. Exercise is a key factor in environmental enrichment, as it triggers a neurochemical interaction that has a significant impact on preventing and treating neurological and mental disorders [19, 20].

Aerobic exercise, when appropriately dosed, enhances the antioxidant system and redox balance, while inappropriate exercise can be detrimental [21]. Exercise influences the mitochondrial respiratory chain, altering oxygen availability, pH, and membrane potential, and calcium levels, thereby affecting ROS production [22, 23, 24]. It also boosts cortical Brain‐derived neurotrophic factor (BDNF) [25], activate the nuclear factor erythroid 2‐related factor 2/peroxisome proliferator‐activated receptor γ Coactivator 1 (NRF2/PGC‐1) pathway, and increases antioxidant enzyme expression like catalase, superoxide dismutase, and glutathione as an adaptive response [26, 27, 28]. Exercise elevates brain nitric oxide (NO) levels, aiding vasodilation and anti‐clotting for improved neuronal blood flow [29]. NO contributes to neuroplasticity via the cyclic guanosine monophosphate (cGMP) pathway [30] and can modulate enzyme activities through nitrosylation, impacting both apoptotic and anti‐apoptotic pathways [31].

Studies highlight that moderate‐intensity continuous training (MICT) can mitigate apoptosis, oxidative stress, and inflammation in brain tissue [32, 33, 34]. MICT enhances brain antioxidant defences, increasing total antioxidant capacity and the activity of enzymes like glutathione peroxidase (GPX), glutathione reductase (GR), superoxide dismutase (SOD) and catalase (CAT), particularly in the cortical area [34, 35]. MICT has been shown to protect against apoptosis in the rat cerebral cortex, reducing apoptotic cells, caspase‐3 expression, while boosting Bcl‐2 and the Bcl‐2/Bax ratio [25, 36]. Gharebaghi et al. demonstrated that treadmill exercise reduces MDMA‐induced lipid peroxidation in rat hippocampus, with exercised rats showing a lower Bax/Bcl‐2 ratio and caspase‐3 expression compared to non‐exercised controls [32].

Given the above, a lifestyle involving regular exercise is crucial to mitigate the psychological and physiological effects of drug abuse [20, 32, 33]. Identifying key biomarkers in pathways linked to oxidative stress and apoptosis from ketamine abuse will inform future recovery strategies. Exercise, as a non‐pharmacological intervention, is vital. This study aimed to assess the effectiveness of MICT in reducing oxidative stress and apoptosis in the cerebral cortex of rats exposed to chronic ketamine.

## 2 Materials and Methods

### 2.1 Animals and Grouping

Twenty‐four mature male Wistar rats (250 ± 20 g, 8 weeks old) were sourced from the Animal Resource Centre at Urmia University (ARCUU). The rats were housed in the Laboratory Animal Services facility under controlled lighting (12‐h light/dark cycle) and provided with ad libitum water and a standard rat diet. All procedures adhered to the ‘Guide for the Care and Use of Laboratory Animals’ as approved by Urmia University's Animal Care and Use Committee (Protocol No: IR‐UU‐AEC‐3/64).After an acclimatisation period of 1 week, the rats were allocated into four groups of six:

1. **Control Group (CON):** No intervention.
2. **Ketamine Abuse Group (KET):** Administered 50 mg/kg/day of ketamine [37, 38] (Alfasan, Utrecht, The Netherlands) via intraperitoneal injection for 8 weeks, followed by immediate euthanasia for tissue sampling.
3. **Ketamine Abuse with Exercise Group (KET + EX):** Treated with 50 mg/kg/day ketamine for 8 weeks, followed by 8 weeks of exercise without ketamine administration, after which tissue samples were taken.
4. **Ketamine Abuse with Withdrawal Group (KET + WD):** Received 50 mg/kg/day ketamine for 8 weeks, then maintained on a sedentary lifestyle without ketamine for another 8 weeks before tissue collection.

This design allowed for the evaluation of ketamine's long‐term effects, withdrawal, and potential amelioration through exercise.

### 2.2 Tissue Sampling

- Upon conclusion of the experimental protocol, the animals were humanely euthanized using an overdose of ketamine (100 mg/kg, Alfasan, Utrecht, The Netherlands). The cortical regions of the brain were then meticulously sectioned using a high‐magnification stereomicroscope (Olympus, Japan) and subsequently rinsed with distilled water. The samples were split into two groups: One segment was dried and preserved at −80°C for 2 weeks, preparing for reverse transcription polymerase chain reaction (RT‐PCR) analysis.
- The other was placed in Bouin's solution for a minimum of 72 h to facilitate subsequent histopathological evaluations.

### 2.3 Exercise Training Programme

One week prior to the start of the exercise training programme, rats were acclimated to treadmill running using a specialised five‐channel rat treadmill (Danesh Yakhte, made in Iran). The MICT programme extended over 8 weeks, conducted in five weekly sessions. Exercise intensity was gauged by each rat's maximum running speed (Vmax) as per established protocols [39].

Each session began with a 10 min warm‐up at 40–50% of Vmax, followed by 20–45 min of main aerobic exercise at 60–75% of Vmax on a flat (zero‐degree incline) treadmill (refer to Table 1 for exact durations). The session concluded with a 5 min cool‐down at 35–40% of Vmax, ensuring gradual recovery and minimising stress on the animals.

**TABLE 1:** Duration of rats' MICT on the treadmill per session per week.

Week numbers	Week 1	Week 2	Week 3 and 4	Week 5 and 6	Week 7 and 8
**Duration**	20 min	25 min	30 min	35 min	45 min

### 2.4 Immunohistochemical Staining

The expression of both pro‐apoptotic and anti‐apoptotic proteins in cerebral cortex tissue was evaluated using immunohistochemical staining (IHC). Tissue sections, cut to thicknesses of 6–5 μm, were prepared from paraffin‐embedded blocks. These sections underwent preheating at 60°C for 25 min, followed by deparaffinization in xylene (twice, 5 min each) and hydration through a series of ethanol concentrations (90%, 80%, 70%, 50% for 3 min each).

Antigen retrieval was performed with a citrate buffer solution (comprising citric acid, sodium citrate, and distilled water, pH 7.2). Endogenous peroxidase activity was then neutralised by a 5 min incubation in 35% hydrogen peroxide. Antigen blocking was achieved using a superblock reagent (Cat No: 237QK100037), followed by overnight incubation (18 h) with primary antibodies from Elabscience, USA (anti‐Bcl‐2, Cat No:E‐AB‐60788, 1:300; anti‐Bax, Cat No: E‐AB‐13814, 1:350; anti‐Caspase‐3, Cat No: E‐AB‐30756, 1:500).

After washing with phosphate buffer saline (PBS, pH 7.2), sections were incubated with a secondary antibody (Goat anti‐rabbit IgG, Cat No: E‐AB1003, 1001). Staining was completed by a 3 min exposure to haematoxylin solution, followed by PBS washing and a 15 min incubation with diaminobenzidine chromogen. The presence of cells positive for Bax, Bcl‐2, caspase‐3, and 8‐oxo‐2′‐deoxyguanosine (OXO) was subsequently analysed [40].

### 2.5 Histological Analyses

After fixation, the cortical brain samples underwent standard processing, which included clearing in xylene and embedding in paraffin wax. These samples were then sectioned at 5–6 μm using a rotary microtome (Historange‐2218, UK). The sections were subsequently stained with haematoxylin and eosin for histological analysis. Examination of the prepared slides was conducted under a light microscope (Olympus‐CH‐2, Japan) [41].

### 2.6 Assessment of Oxidative Stress and Redox Markers

The homogenisation of cortical tissue was carried out using 10 volumes (1:10, w/v) of a 20 mM sodium phosphate buffer (pH 7.4) supplemented with 140 mM KCl. Following centrifugation at 800 × g for 10 min at 4°C, the resulting pellet was discarded, and the supernatant was utilised for the assessment of oxidative stress markers according to the methodology described by Schmitz et al [42].

#### 2.6.1 Malonaldehyde Concentrations

Malondialdehyde (MDA) levels, indicative of lipid peroxidation, were quantified using the thiobarbituric acid (TBA) assay. The Navand Salamat Lipid Peroxidation (MDA) Assay Kit (Nolandi) from Iran was utilised. Tissue homogenates were combined with TBA reagent as per the manufacturer's instructions to form chromogenic products. After centrifugation, the absorbance of the supernatants was measured at 532 nm, and MDA concentrations were calculated using a standard curve.

#### 2.6.2 Myeloperoxidase Concentrations

The total activity of Myeloperoxidase (MPO) in serum was measured using the enzyme‐linked immunosorbent assay (ELISA) method. The Navand Salamat Myeloperoxidase (MPO) Activity Assay Kit (Nampox) from Iran was utilised. The assay was conducted following the manufacturer's instructions, and an ELISA plate reader was used to measure absorbance at a wavelength of 650 nm.

#### 2.6.3 SOD Activity

SOD activity was evaluated by its ability to inhibit the autoxidation of pyrogallol, measured spectrophotometrically at 420 nm, where SOD's substrate, superoxide anion (O₂^−^), plays a crucial role. The Navand Salamat Superoxide Dismutase (SOD) activity Assay Kit (Nasdox) from Iran was utilised. The assay mixture comprised 50 mM Tris buffer (pH 8.2), 1 mM EDTA, 80 U/mL catalase, 0.80 mM pyrogallol, and tissue supernatant containing approximately 45 μg of protein. The enzyme's specific activity was quantified in units per milligramme of protein (U/mg) using a calibration curve with purified SOD [43].

#### 2.6.4 CAT Activity

CAT activity was assessed by measuring the decomposition of hydrogen peroxide (H₂O₂) at 240 nm. The reaction mixture contained 20 mM H₂O₂, 0.1% Triton X‐100, 10 mM potassium phosphate buffer (pH 7.0), and tissue protein at concentrations of 0.1–0.3 mg/mL. The Navand Salamat Catalase Activity Assay Kit (Nactaz) from Iran was utilised. The activity was expressed in units per milligramme of protein, where one unit of CAT activity corresponds to the degradation of 1 μmol of H₂O₂ per minute [44].

#### 2.6.5 NO Production

In summary, the accumulation of nitrite (NO₂^−^) in the culture medium was measured by combining an equal volume of the medium with Griess reagent and incubating it at room temperature for 15 min. The absorbance at 540 nm, indicative of azo dye formation, was then quantified using an ultraviolet–visible spectrophotometer (SP‐8001, Metertech, Taipei, Taiwan). Sodium nitrite served as the standard for calibration [45].

#### 2.6.6 Glutathione Levels

The NarGul‐Glutathione Assay Kit‐GSH was employed to quantify total Glutathione (GSH) in rat cerebral cortex. The cerebral cortex homogenate was first prepared as described earlier. Deproteinization was achieved by adding an equal volume of 2.5% (w/v) metaphosphoric acid to the homogenate, followed by centrifugation at 10 000 rpm to obtain the supernatant. This supernatant was then neutralised with 5 μL of 4 M triethanolamine per 100 μL. Fifty microliters of this treated cortex supernatant was used for the assay. The GSH concentration was determined via a linear regression analysis, generating a standard curve from 0 to 10 μM. The total GSH content was expressed as nanomoles per microgram of cerebral cortex protein.

### 2.7 Terminal Deoxynucleotidyl Transferase dUTP Nick End Labelling (TUNEL) Staining

To assess the apoptosis index, we utilised the TUNEL staining kit (in situ cell death detection kit, POD, Roche; Cat No: 11684817910, Germany). Sections of 5–6 μm thickness were deparaffinised in xylene for three minutes each, three times. The slides were rehydrated through a descending ethanol series (96% to 70%, three minutes each) and rinsed with distilled water. They were then incubated with 10–20 μg/mL proteinase K (in 10 mM Tris/HCl, pH 7.4–8) at 37°C for 15 min, followed by three washes in phosphate‐buffered saline (PBS).

Next, sections were treated with 25 μL of TUNEL reaction mixture for 60 min at 37°C, followed by PBS rinses. The slides were then incubated with 25 μL of POD‐converter solution at 37°C for 30 min and washed three times with PBS. Visualisation was achieved by applying approximately 25 μL of 3,3′‐diaminobenzidine substrate for 60 s, after which the sections were washed with distilled water three times.

The apoptosis index was determined by analysing 20 transverse epithelial sections per animal, using the formula: 100 × (number of TUNEL‐positive cell nuclei/total number of cell nuclei).

### 2.8 RNA Isolation, cDNA Synthesis, and qRT‐PCR

Total mRNA was extracted from cerebral cortex tissues, previously stored at −80°C, using a TRIzol‐based extraction kit (Sina‐Gen, Tehran, Iran). The RNA concentration was quantified at 260 nm using a NanoDrop spectrophotometer, and its purity was assessed by the A260/280 ratio. Samples with an A260/280 ratio between 1.8 and 2.0 were selected for further processing.

cDNA synthesis was carried out in a 20 μL reaction mixture containing 1 μg RNA, 1 μL oligo (dT) primer, 4 μL 5 × reaction buffer, 1 μL RNAse inhibitor, 2 μL 10 mM dNTP mix, and 1 μL M‐MuLV Reverse Transcriptase, following the manufacturer's protocol. The thermal cycling conditions were: 65°C for 5 min, 47°C for 60 min, and 70°C for 5 min.

For qRT‐PCR, a 27 μL reaction volume was prepared, including 13 μL PCR master mix, 1 μL each of forward and reverse primers for the specific genes (Bcl‐2, Bax, Caspase‐3, and GAPDH as the housekeeping gene), 1.5 μL cDNA template, and 10.5 μL nuclease‐free water. The thermal cycling protocol included an initial denaturation at 95°C for 3 min, followed by 35 cycles of: 95°C for 20 s, annealing at 62°C for Bcl‐2 (1 min), 59°C for Bax (1 min), 50°C for Caspase‐3 (45 s), and 60°C for GAPDH (45 s), with a final extension at 72°C for 1 min, ending with 72°C for 5 min.

The resulting PCR products were visualised on a 1.5% agarose gel and analysed using PCR Gel analysing software (ATP, Tehran, Iran). Band intensities were compared to controls set at 100 for normalisation purposes.

### 2.9 Statistical Analyses and Imaging

Tissue images were captured and processed using Adobe Photoshop CC 2018 in conjunction with an optical microscope equipped with a Sony Cyber‐Shot camera (Japan). To assess the normality of data and homogeneity of variances, the Shapiro–Wilk (S‐W) and Levene's (LVN) tests were respectively applied. All quantitative data were analysed using SPSS software (version 26.0, California, USA), employing one‐way ANOVA followed by Tukey's multiple comparison test for post hoc analysis. Statistical significance was set at a *p*‐value less than 0.05 (*p* < 0.05). Effect sizes (ES) were computed to evaluate the magnitude of differences among groups. Figures were generated with GraphPad Prism version 10.1.0 (California, USA). Data are presented as mean ± standard deviation.

## 3 Results

### 3.1 Histopathological Analysis

**FIGURE 1:**
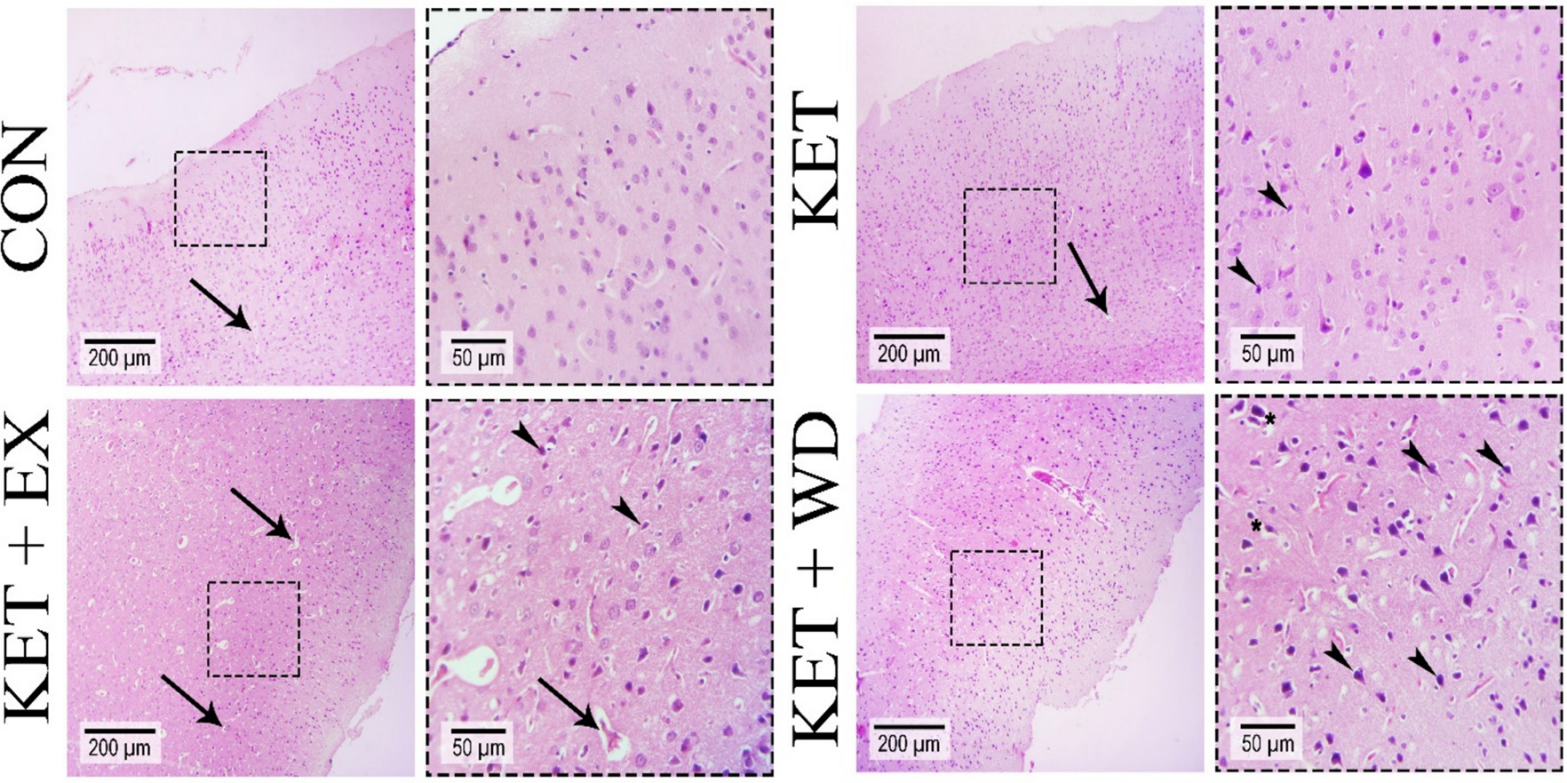
Photomicrographs from the cerebral cortex of the brain tissue (H & E staining); arrow: vasogenic edema, arrowhead: pyknotic nuclei, asterisk: perineuronal edema. CON: Control, KET: Ketamine, KET + EX: Ketamine + Exercise, KET + WD: Ketamine + Withdrawal.

Histopathological examination of cerebral cortex photomicrographs (Figure 1) revealed the following:

- *CON Group:* Minimal pyknotic nuclei, with moderate vasogenic edema (indicated by arrows) and low perineuronal edema were noted.
- *KET Group:* Displayed a moderate to high incidence of pyknotic nuclei (indicated by arrowheads), high vasogenic edema (arrows), and moderate perineuronal edema.
- *KET + EX Group:* Showed a large number of pyknotic nuclei (arrowheads), high vasogenic edema (arrows), and moderate perineuronal edema.
- *KET + WD Group:* Exhibited severe pyknotic nuclei (arrowheads), vasogenic edema (arrows), and perineuronal edema (asterisks).

For comprehensive details, refer to Table 2.

**TABLE 2:** Average damage rate of the cerebral cortex per group.

Damage type	Groups*			
	CON	KET	KET + EX	KET + WD
Pyknotic nuclei	1.66	2.33	3	3.66
Vasogenic edema	2	2.66	2.66	3.33
Perineuronal edema	1.6	2	2.33	3.33

* Score 1 + indicating low damage rate (0–25% of tissue), score 2 + indicating moderate damage rate (26–50% of tissue), score 3 + indicating high damage rate (51–75% of tissue) and, score 4 + indicating severe damage rate (76–100% of tissue).

### 3.2 MICT Decreased Bax, and Caspase‐3 mRNA Expression

**FIGURE 2:**
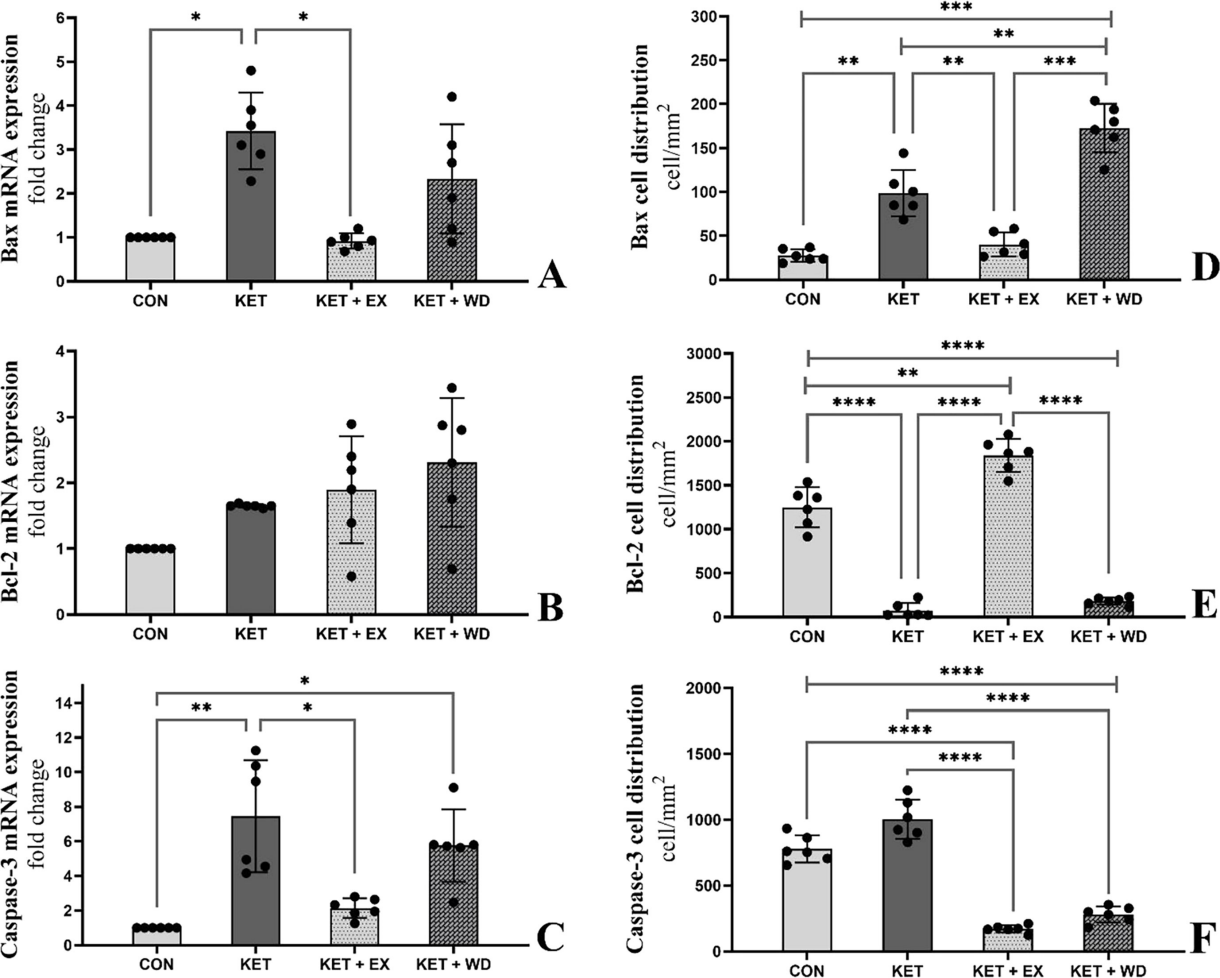
(A), (B), and (C) showing RT‐PCR electrophoresis gel results respectively for Bax, Bcl‐2, and caspase‐3. (D), (E), and (F) showing cell distribution per mm^2^ of tissue respectively for Bax, Bcl‐2, and caspase‐3. CON: Control, KET: Ketamine, KET + EX: Ketamine + Exercise, KET + WD: Ketamine + Withdrawal.

We assessed the mRNA expression levels of Bax (Figure 2A), Bcl‐2 (Figure 2B), and caspase‐3 (Figure 2C), which are central regulators of mitochondria‐dependent apoptosis. Homogeneity of variance (HOV) and normality of data (NOD) tests for these mRNA expressions confirmed that the assumptions were met (Shapiro–Wilk: *p* > 0.05, Levene's: *p* > 0.05).

The results indicated a significant elevation in both Bax (ES = 3.93, *p* = 0.016) and caspase‐3 (ES = 2.82, *p* = 0.006) mRNA levels in the KET group compared to the CON group. However, following the intervention with MICT, there was a significant reduction in Bax (ES = 4.00, *p* = 0.013) and caspase‐3 (ES = 2.29, *p* = 0.024) mRNA levels. No significant differences were observed in the mRNA expression of Bcl‐2 across the groups.

### 3.3 MICT Decreased the Levels of Bax and Caspase‐3 Proteins While Increasing the Levels of Bcl‐2 Proteins

**FIGURE 3:**
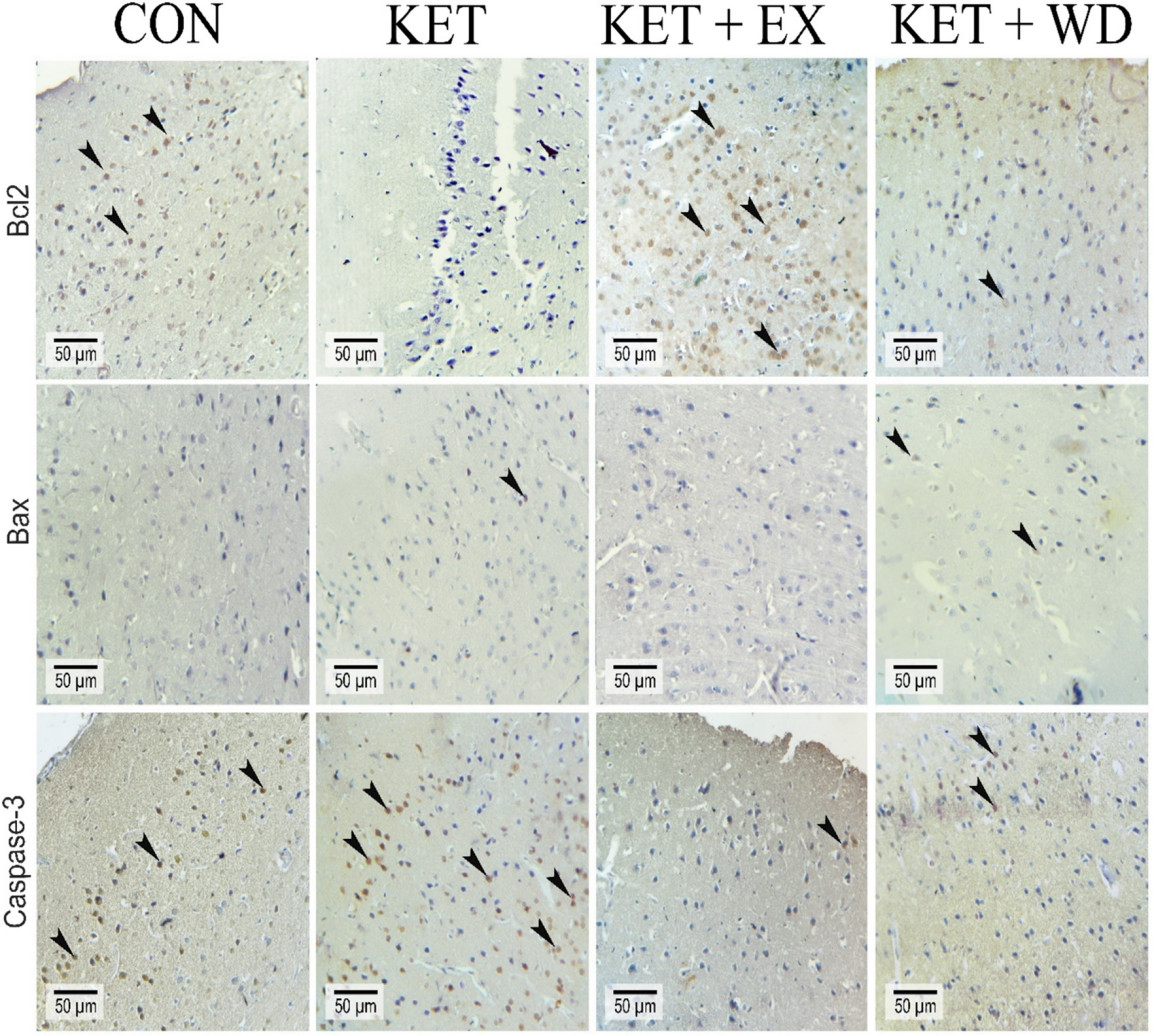
Immunohistochemical staining for Bcl‐2, Bax, and caspase‐3; positive reactions for proteins are indicated by arrowheads in different groups. CON: Control, KET: Ketamine, KET + EX: Ketamine + Exercise, KET + WD: Ketamine + Withdrawal.

The cellular distribution of Bax (Figure 2D), Bcl‐2 (Figure 2E), and caspase‐3 (Figure 2F) was assessed. We further analysed the immunohistochemical staining (Figure 3) and conducted a pixel‐based histogram analysis (Figure 5) for these markers. The tests for homogeneity of variance (HOV) and normality of data (NOD) were performed for the protein expressions of Bax, Bcl‐2, and caspase‐3 (S‐W: *p* > 0.05, LVN: *p* > 0.05).

Ketamine treatment led to an increase in Bax protein levels (ES = 3.67, *p* = 0.001) when compared to the control group. MICT significantly reduced the protein levels of both Bax (ES = 2.77, *p* = 0.005) and caspase‐3 (ES = 7.73, *p* < 0.0001) relative to the KET group. The caspase‐3 protein level in the KET + WD group was notably higher than in the CON group. Ketamine administration also resulted in a significant reduction in Bcl‐2 protein levels (ES = 6.86, *p* < 0.0001), but in the KET + EX group, Bcl‐2 protein levels were increased compared to both the KET (ES = 12.11, *p* < 0.0001) and KET + WD (ES = 12.14, *p* < 0.0001) groups.

#### 3.3.1 MICT Decreased TUNEL Positive Cells

**FIGURE 4:**
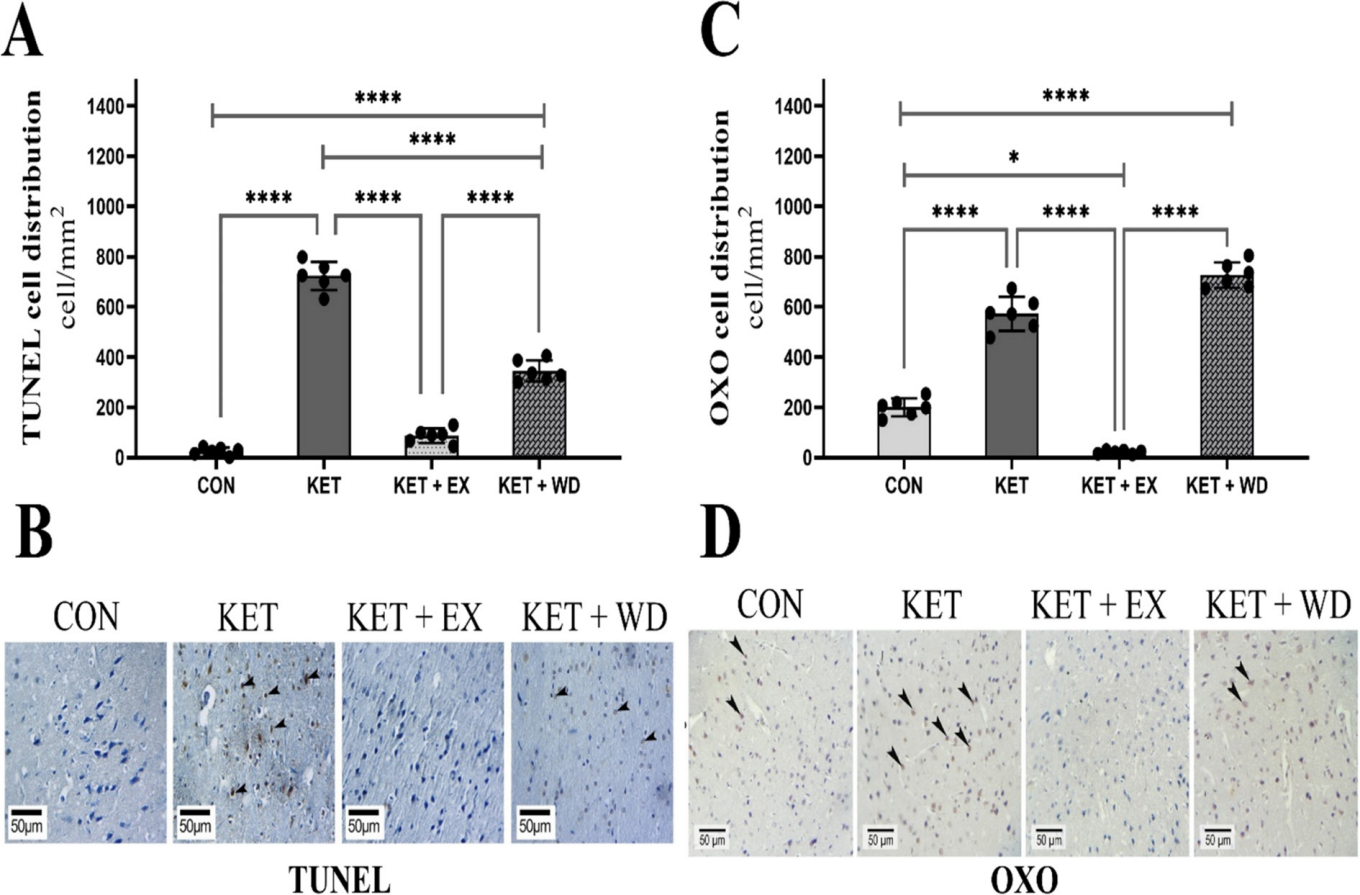
(A) and (C) showing cell distribution per mm^2^ of tissue respectively for TUNEL and OXO. (B) and (D) showing immunohistochemical staining for TUNEL and OXO; positive reactions for proteins are indicated by arrowheads in different groups. CON: Control, KET: Ketamine, KET + EX: Ketamine + Exercise, KET + WD: Ketamine + Withdrawal.

The S‐W and LVN tests confirmed that the data for TUNEL cell distribution were normally distributed and had homogeneous variances (*p* > 0.05). TUNEL staining revealed a significant increase in apoptotic cells in the KET group compared to the CON group (ES = 16.99, *p* < 0.0001). The KET + EX group showed a marked reduction in TUNEL‐positive cells relative to the KET group (ES = 14.24, *p* < 0.0001). (Figure 4A,B).

#### 3.3.2 MICT Decreased DNA Oxidation

**FIGURE 5:**
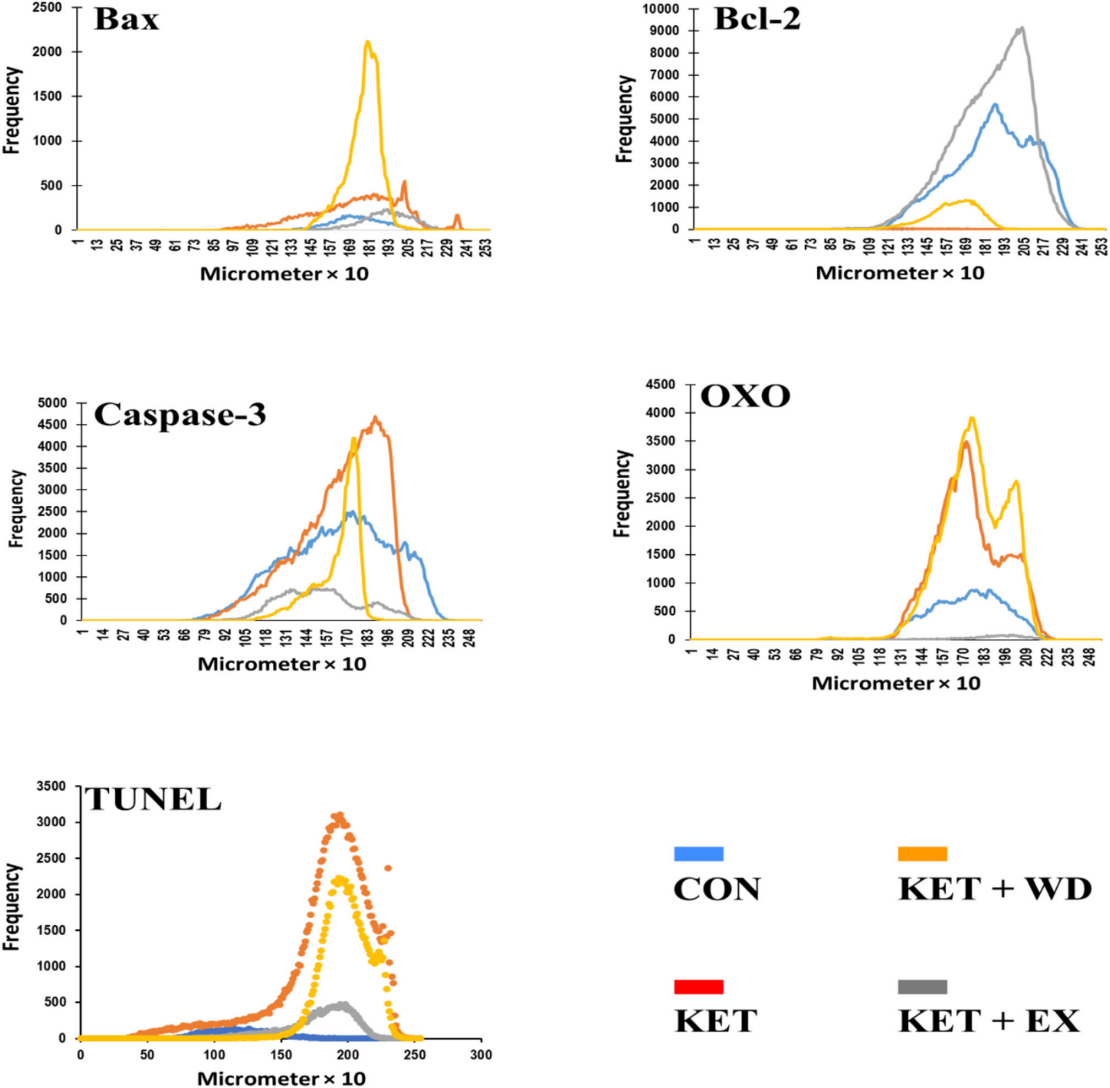
Pixel‐based histogram analysis for Bax, Bcl‐2, caspase‐3, OXO, and TUNEL positive reactions in 253 × 10 μm of one cross‐section. CON: Control, KET: Ketamine, KET + EX: Ketamine + Exercise, KET + WD: Ketamine + Withdrawal.

The IHC staining, cell distribution, and pixel‐based histogram (Figure 5) analysis for OXO were assessed and normality of data and homogeneity of variances were confirmed (S‐W: *p* > 0.05, LVN: *p* > 0.05). IHC analyses indicated a significant elevation in OXO levels in both the KET (ES = 6.85, *p* < 0.0001) and KET + WD (ES = 11.96, *p* < 0.0001) groups compared to the CON group. However, MICT significantly lowered OXO levels when compared to the KET group (ES = 11.39, *p* < 0.0001). (Figure 4C,D).

#### 3.3.3 MICT Increased GR and GPX mRNA Expression

We evaluated the mRNA expressions of GR and GPX as antioxidant markers. The S‐W and LVN tests confirmed that the data for GR and GPX mRNA expressions were normally distributed and had homogeneous variances (*p* > 0.05). The KET group did not show a significant reduction in GPX or GR mRNA expression compared to the CON group. However, MICT significantly enhanced the expression of both GR (ES = 2.02, *p* = 0.016) and GPX (ES = 1.98, *p* = 0.035) mRNA when compared to the KET group. Notably, there were significant increases in GR mRNA expression in the KET + EX group relative to both the CON (ES = 3.39, *p* = 0.01) and KET + WD (ES = 2.82, *p* = 0.008) groups. (Table 3).

**TABLE 3:** Glutathione peroxidase (GPX) and glutathione reductase (GR) mRNA expression levels.

Variables	Groups				*p*
	CON	KET	KET + EX	KET + WD	
GPX mRNA (Fold change)	1	0.36 ± 0.46	1.15 ± 0.76^**b**^	0.91 ± 0.54	0.21
GR mRNA (Fold change)	1	1.09 ± 0.66	2.32 ± 0.55^a^, ^b^, ^c^	0.95 ± 0.41	0.004

Abbreviations: CON: Control, KET: Ketamine, KET + EX: Ketamine + Exercise, KET + WD: Ketamine + Withdrawal.

^a^ indicates significant differences compared to CON.

^b^ indicates significant differences compared to KET.

^c^ indicates significant differences compared to KET + WD.

*Note:* Data are presented as Mean ± SD.

#### 3.3.4 MICT Had No Effect on MDA, GSH, CAT, SOD, and MPO Levels

The LVN and S‐W tests confirmed the homogeneity of variances and the normality of data for CAT, SOD, GSH, MDA, MPO, and NO (*p* > 0.05). No significant differences were found in CAT, SOD, or MPO activity across all groups (*p* > 0.05). Similarly, there were no significant variations in MDA and GSH levels among the groups (*p* > 0.05). (Table 4).

**TABLE 4:** Oxidative stress and redox activity or levels after the intervention.

Variables	Groups				*p*
	CON	KET	KET + EX	KET + WD	
GSH (μm)	30.38 ± 0.85	29.73 ± 0.82	33.25 ± 3.49	29.26 ± 0.59	0.11
CAT (nmol/min/mg pro)	9.41 ± 0.84	9.93 ± 1.16	9.21 ± 0.15	9.77 ± 0.44	0.65
SOD (U/mg pro)	506.25 ± 35.44	505.03 ± 18.43	499.61 ± 19.27	433.61 ± 86.61	0.27
NO (μm)	104.01 ± 6.16	58.64 ± 30.23	150.35 ± 20.97^**a**^	165.13 ± 32.28b	0.003
MDA (nmol/mg pro)	18.30 ± 1.67	18.82 ± 1.21	19.68 ± 1.69	17.23 ± 0.98	0.27
MPO (U/g tissue)	1213.7 ± 126.48	1290.6 ± 446.39	1012.8 ± 122.29	1324.8 ± 116.33	0.45

Abbreviations: CON: Control, KET: Ketamine, KET + EX: Ketamine + Exercise, KET + WD: Ketamine + Withdrawal. GSH: Glutathione, CAT: Catalase, SOD: Superoxide dismutase, NO: Nitric Oxide, MDA: Malondialdehyde, MPO: Myeloperoxidase.

*Note:* Data are presented as Mean ± SD.

^a^ indicating significant differences compared to the KET group (*p* < 0.05).

#### 3.3.5 MICT and Ketamine Withdrawal Increased NO Protein Level.

There were no significant differences in NO levels between the CON and KET groups. However, NO levels significantly increased in both the KET + EX (ES = 3.52, *p* < 0.001) and KET + WD (ES = 3.40, *p* < 0.001) groups compared to the KET group. No significant differences were observed between the KET + EX and KET + WD groups (*p* > 0.05). (Table 4).

## 4 Discussion

To our knowledge, no study has explored how exercise training affects brain apoptosis and oxidative stress due to chronic ketamine abuse. This study aimed to assess the impact of 8 weeks of MICT on apoptosis and oxidative stress indicators in the rat cerebral cortex. MICT reduced TUNEL‐positive cells, and decreased mRNA and protein levels of Bax and Caspase‐3 in ketamine‐exposed rats. MICT could increase Bcl‐2 protein level in cerebral cortex compare to KET and KET + WD groups. However, no such reduction was seen in the ketamine withdrawal (KET + WD) group. Histologically, ketamine caused cortical neuron damage, which persisted post‐withdrawal, but MICT mitigated further cell damage progression. Sun and colleagues, consistent with our findings, showed that long‐term ketamine abuse in monkeys increased TUNEL‐positive cells and the expression of Bax and Caspase‐3, while decreasing Bcl‐2 in the cerebral cortex [46]. Our findings align with studies showing aerobic exercise reduces apoptosis in brain tissue due to drug abuse [47, 48, 49]. Gharebaghi et al. found treadmill exercise significantly decreased caspase‐3 and Bax/Bcl‐2 in ecstasy‐treated rat hippocampus [32]. Mokhtari Zaer et al. demonstrated that voluntary treadmill exercise increased Bcl‐2, counteracting daily morphine's effects, while reducing Bax and withdrawal symptoms [50].

Several drugs are known to increase glutamate release and initiate neuronal apoptosis [51, 52, 53, 54, 55]. This glutamate surge leads to calcium ion influx via NMDA receptor activation, triggering apoptosis and elevating ROS [56, 57, 58]. Chronic ketamine abuse similarly boosts extracellular glutamate and neuronal apoptosis by causing a persistent and irreversible increase in intracellular calcium [59, 60, 61]. While some studies propose that ketamine‐induced calcium elevation might be a compensatory mechanism not directly tied to drug concentration in blood or brain, others argue it results from direct ketamine action on NMDA receptors and extracellular calcium influx [62].

Exercise training activates neurotrophic factors signalling pathways that reduce brain apoptosis, with BDNF being extensively studied in this context. Both high‐ and moderate‐intensity exercise training have been shown to elevate BDNF levels in serum and brain tissue [27, 63, 64]. Increased BDNF can trigger protective signalling pathways [65], including inhibiting apoptosis through the hedgehog pathway and enhancing erythropoietin levels [66]. BDNF also blocks apoptosis by boosting Bcl‐2 protein, NO production, cGMP, and Protein Kinase G 1 gene expression. Our study suggests that MICT exerts its anti‐apoptotic effects by modulating these pathways, particularly by increasing NO levels.

We showed that MICT can improve the redox state and reduce DNA oxidation in the cerebral cortex post‐ketamine exposure. In the present study, MICT increased GR and GPX mRNA expression. However, levels and activity of markers such as MDA, MPO, GSH, CAT, and SOD remained unchanged following ketamine use, exercise, or withdrawal. In contrast to our results, Zhang et al. noted that 12 weeks of aerobic training in methamphetamine addicts boosted total antioxidant capacity and CAT activity, with no change in SOD levels [67]. Gharebaghi et al. showed treadmill exercise reduces ecstasy‐induced lipid peroxidation in rat hippocampus [32]. Chalimoniuk et al. found that 6 weeks of moderate endurance training in healthy rats increased SOD and CAT activity only in the neocortex, with no change in GPX [35]. Notably, in our study, MICT raised NO levels in the KET + EX group compared to the KET group, reducing DNA damage (OXO). NO has a dual nature: protective under normal conditions but neurotoxic during oxidative stress due to agents like peroxynitrite and cyclooxygenase [68]. Oleson et al. highlighted NO's dual roles, noting that in pancreatic beta cells, NO both impairs function and reduces apoptosis by preventing DNA damage, thus acting protectively. However, they observed no such DNA damage reduction in macrophages, hepatocytes, or fibroblasts, indicating this behaviour is specific to pancreatic beta cells [69]. This study found that MICT elevates NO levels while reducing DNA damage and apoptosis, suggesting a protective role for NO in cerebral cortex neurons. The exact mechanisms warrant further investigation.

The lack of change in SOD, CAT, GSH, MDA, and MPO levels observed in this study, despite clear evidence of apoptosis and DNA damage in the cerebral cortex caused by chronic ketamine abuse, presents an intriguing phenomenon. One possible explanation is that ketamine‐induced ROS production might be regionally specific, affecting intracellular oxidative stress markers more than systemic cerebral markers. Additionally, ketamine‐induced oxidative damage may bypass the antioxidant systems measured in this study, directly initiating apoptosis via alternative pathways [70, 71] such as NMDA receptor activation and calcium influx, which are known to trigger mitochondrial dysfunction and ROS production independently of the traditional antioxidant defence system [72]. However, the nuanced interaction between exercise, ketamine‐induced ROS, and the antioxidant defence system warrants further exploration, particularly focusing on intracellular and region‐specific oxidative stress markers and their interplay with apoptosis signalling pathways.

### 4.1 Limitations

The main limitations of our study include:

- Not evaluating markers in the BDNF/NRF2/PGC‐1 signalling pathway concerning ketamine abuse and exercise training.
- Lack of analysis on NO nitrosylation, its effects on ROS, and apoptosis.
- Not assessing the impact of ketamine and exercise on oxidative status and apoptosis in other brain regions like the hippocampus.
- Inability to present mRNA data visually due to imaging technology constraints.

## 5 Conclusion

Our findings show that MICT reduces apoptotic cells and apoptosis marker expression in the cerebral cortex of rats with ketamine abuse. It also increases GR and GPX gene expression and NO levels while decreasing DNA oxidation. This suggests MICT could help ameliorate the damage from ketamine abuse, aiding recovery.

## Conflicts of Interest

The authors declare no conflicts of interest, financial or otherwise.

## Data Availability

The data that support the findings of this study are available from the corresponding author upon reasonable request.
